# Comparison of neurons derived from mouse P19, rat PC12 and human SH-SY5Y cells in the assessment of chemical- and toxin-induced neurotoxicity

**DOI:** 10.1186/s40360-017-0151-8

**Published:** 2017-06-05

**Authors:** Dina Popova, Jessica Karlsson, Stig O. P. Jacobsson

**Affiliations:** Department of Pharmacology and Clinical Neuroscience, Umeå University, Umeå, Sweden

**Keywords:** In vitro cytotoxicity, Neuronal cell cultures, Retinoic acid-treated P19 cells, Retinoic acid-treated SH-SY5Y cells, Nerve growth factor-treated PC12 cells, Neurotoxicity, Methylmercury, Okadaic acid, Acrylamide, Glutathione

## Abstract

**Background:**

Exposure to chemicals might be toxic to the developing brain. There is a need for simple and robust in vitro cellular models for evaluation of chemical-induced neurotoxicity as a complement to traditional studies on animals. In this study, neuronally differentiated mouse embryonal carcinoma P19 cells (P19 neurons) were compared with human neuroblastoma SH-SY5Y cells and rat adrenal pheochromocytoma PC12 cells for their ability to detect toxicity of methylmercury (MeHg), okadaic acid and acrylamide.

**Methods:**

Retinoic acid-treated P19 and SH-SY5Y cells and nerve growth factor-stimulated PC12 cells, allowed to differentiate for 6 days, were exposed to MeHg, okadaic acid and acrylamide for 48 h. Cell survival and neurite outgrowth were assessed with the calcein-AM assay and fluorescence detection of antibodies against the cytoskeletal neuron-specific protein βIII-tubulin, respectively. The effects of glutathione (GSH) and the potent inhibitor of GSH synthesis buthionine sulfoximine (BSO) on the MeHg induced-toxicity were assessed using the PrestoBlue™ cell viability assay and the TMRE mitochondrial membrane potential assay.

**Results:**

Differentiated P19 cells developed the most extensive neuronal network among the three cell models and were the most sensitive neuronal model to detect neurotoxic effects of the test compounds. MeHg produced a concentration-dependent toxicity in differentiated P19 cells and SH-SY5Y cells, with statistically significant effects at concentrations from 0.1 μM in the P19 neurons and 1 μM in the SH-SY5Y cells. MeHg induced a decrease in the cellular metabolic activity and mitochondrial membrane potential (ΔΨm) in the differentiated P19 cells and SH-SY5Y cells, that were attenuated by GSH. Okadaic acid and acrylamide also showed statistically significant toxicity in the P19 neurons, but not in the SH-SY5Y cells or the P12 cells.

**Conclusions:**

P19 neurons are more sensitive to detect cytotoxicity of MeHg, okadaic acid and acrylamide than retinoic acid-differentiated SH-SY5Y cells and nerve growth factor-treated PC12 cells. P19 neurons are at least as sensitive as differentiated SH-SY5Y cells to detect the loss of mitochondrial membrane potential produced by MeHg and the protective effects of extracellular GSH on MeHg toxicity. P19 neurons may be a useful model to study neurotoxic effects of chemicals.

## Background

Humans are exposed to a growing number of chemicals, including environmental pollutants, food preservatives, cosmetics and drugs which might be potentially toxic to the human health. Traditional toxicological studies performed on animals are time-consuming, expensive and do not yield mechanistic information. There is a need for simple and robust in vitro cellular models that allow a rapid toxicological screening of a large number of chemicals. Moreover, cellular models are useful to study specific mechanisms of chemical-induced toxicity as a complement to more complex investigations on animals [1]. With respect to in vitro models for neurotoxicity studies, a balance needs to be struck between the complexity of the model and its capacity, since there is inevitably a reciprocal relationship between these two considerations. We have focussed upon cellular models with sufficient capacity to allow for detailed mechanistic investigations. In this respect, rat pheochromocytoma PC12 cells and human neuroblastoma SH-SY5Y cells are simple and yet elegant in vitro models for neurotoxicity studies [2, 3].

PC12 cells originate from a rat adrenal pheochromocytoma isolated in 1976. Upon exposure to nerve growth factor (NGF), PC12 cells undergo neuronal differentiation [4]. NGF-treated PC12 cells release several neurotransmitters including dopamine [5], noradrenaline [6] and acetylcholine [7].

The human cell line SH-SY5Y is a subclone of SK-N-SH, which was isolated in 1970 from the bone marrow aspiration of a 4-year-old girl with metastatic neuroblastoma [8]. When stimulated with retinoic acid (RA), SH-SY5Y differentiates to dopaminergic neuron-like cells [9].

P19 cells were isolated in 1981 from a mouse teratocarcinoma [10]. The P19 cell line is multipotent, it can differentiate into derivates from all three germ layers: endoderm, mesoderm, and neuroectoderm. Upon exposure to RA, P19 differentiate predominantly into neuronal-like cells (P19 neurons), but also astroglia and fibroblast-like cells [11]. P19 neurons are postmitotic and possess functional synapses [12, 13]. Several excitatory and inhibitory neurotransmitters and associated receptors and enzymes have been detected in P19 neurons, including glutamate, GABA, and acetylcholine [14–19].

In this study, neuronally differentiated mouse embryonal carcinoma P19 cells have been compared with human neuroblastoma SH-SY5Y cells and rat pheochromocytoma PC12 cells for their ability to respond to the toxicity of methylmercury (MeHg), okadaic acid and acrylamide.

MeHg is an organic form of the heavy metal mercury. Mercury is transformed to MeHg in bacteria [20] and bioaccumulates in fish [21]. The primary route of human exposure is consumption of seafood. MeHg is highly toxic to the central nervous system and particularly to the developing brain [22]. One of the key targets of MeHg toxicity is the glutathione (GSH) antioxidant system [23–25]. In mouse, in utero exposure to MeHg leads to reduced GSH levels and inhibits the activity of glutathione peroxidase and glutathione reductase in the developing brain [25]. In human neurons, astrocytes and SH-SY5Y cells, extracellular treatment with GSH protects against MeHg-induced cytotoxicity. Moreover, buthionine sulfoximine (BSO), a potent inhibitor of GSH synthesis [26] increases MeHg-induced toxicity in these cell types [24].

Another key target of MeHg cytotoxicity is the mitochondria. MeHg induces mitochondrial dysfunctions in different neuronal cell types and tissues [27–30], among them loss of mitochondrial membrane potential (Δψm) has been detected in mouse hippocampal HT22 cell line [27] and neonatal rat primary cerebellar granule cells [28].

MeHg inhibits microtubule polymerization in the cytoskeleton in vitro [31, 32], an important process for maintaining cell structure and viability [33]. MeHg also inhibits neurite outgrowth and causes neurite degeneration in studies on animals and in in vitro cellular models [2, 34–36].

Okadaic acid is a marine biotoxin responsible for diarrheal shellfish poisoning. It is a potent inhibitor of protein phosphatases [37]. Neurotoxicity of this compound has been observed both in in vitro [38, 39] and in vivo studies [40, 41].

Acrylamide is widely used in the industry over the world and it also forms naturally in starch containing food cooked at high temperatures [42]. Studies on animals [43, 44] and in cellular models [3] have shown neurotoxicity of acrylamide.

In the present study, we have compared the sensitivity of the three described cell lines towards MeHg, okadaic acid and acrylamide during the process of neuronal development. The use of the three cell lines allows delineation of both common and cell-specific neurotoxic mechanisms for a given compound.

The effects of the chemicals on cell survival and neurite outgrowth were investigated. Cell viability was assessed with the calcein-AM assay that measures intracellular esterase activity. Neurite outgrowth was assessed using immunocytochemical labeling of βIII-tubulin, a component of microtubules in the cytoskeleton specific for nerve cells [45]. In addition, the role of GSH on MeHg-induced effects on cellular metabolic reduction, extracellular lactate dehydrogenase (LDH) release and mitochondrial membrane potential were assessed in RA-treated P19 and SH-SY5Y cells.

## Methods

### Chemicals

Dulbecco’s modified Eagle’s medium with high glucose (DMEM), minimal essential medium with Earl’s salts (EMEM), Dulbecco’s modified medium with Ham’s F12 medium (DMEM/F12), poly-D-lysine hydrobromide, dimethyl sulfoxide (DMSO), all-trans retinoic acid (RA), rat nerve growth factor β (NGFβ), okadaic acid, methylmercury (II) chloride, acrylamide, DL-Buthionine-[S,R]-sulfoximine (BSO), L-glutathione reduced (GSH), tetramethylrhodamine ethyl ester perchlorate (TMRE) and bovine serum albumin were purchased from Sigma-Aldrich (Stockholm, Sweden). MEM-α medium containing deoxyribonucleosides and ribonucleosides (αMEM), neurobasal medium, B27 supplement, N2 supplement, fetal bovine serum (FBS), L-glutamine, non-essential amino acids (NEAA), trypsin/EDTA solution, heat-inactivated horse serum (HS), penicillin/streptomycin (PEST), trypan blue, Alexa Fluor 488 goat anti-rabbit antibodies, Hanks’ balanced salt solution (HBSS) with CaCl_2_ and MgCl_2_, PrestoBlue™ cell viability reagent and calcein-AM were purchased from Invitrogen Life Technologies (Uppsala, Sweden). Triton X-100 and EDTA were obtained from MERCK (Darmstadt, Germany). Purified polyclonal rabbit antibody βIII-tubulin was purchased from Convance Inc. (Princeton, New Jersey, USA). Cytotoxicity detection kit (LDH) was obtained from Roche Diagnostics (Mannheim, Germany). Formaldehyde solution (4%) was purchased from Apotek Produktion & Laboratorier (Umea, Sweden).

### Cell culture and neuronal differentiation

P19 mouse embryonal carcinoma cells (cat. no. ECACC 95102107), PC12 rat adrenal pheochromocytoma cells (cat. no. ECACC 88022401) and SH-SY5Y human neuroblastoma cells (cat. no. ECACC 94030304) were obtained from European Collection of Authenticated Cell Cultures (ECACC) (Porton Down, UK).

P19 cells were cultured in αMEM medium (10% FBS, 1% NEAA, 1 IU/ml penicillin and 1 μg/ml streptomycin) in T75 flasks. The cultures were split 1:20–1:100 at 70–80% of confluence using 0.05% trypsin/0.02% EDTA solution in PBS (3 min incubation at 37 °C, 5% CO_2_).

The neuronal differentiation was induced according to the protocol of Yao et al. [19]. The cells were maintained in Neurobasal medium containing B27 supplement as reported in Svensson et al. [18]. The process of neuronal differentiation of P19 cells included two steps: induction with retinoic acid (RA) (4 days) and differentiation up to 10 days. The cells were plated on a bacterial-grade petri dish (Ø92 mm, Sarstedt Inc., Newton, NC) (1 × 10^6^ cells/dish) in αMEM medium (5% FBS, 1% NEAA, 1 IU/ml penicillin and 1 μg/ml streptomycin). RA (1 μM) was added to induce the process of differentiation. The medium was replaced after 48 h. After totally 96 h of induction with RA, the cells (500 cells/mm^2^) were plated on poly-D-lysine coated (0.05 mg/ml) black clear bottom 96-well plates in Neurobasal medium containing 2% B27 supplement, 0.5 mM L-glutamine, 1 IU/ml penicillin and 1 μg/ml streptomycin. Half of the medium/well was replaced every second day.

PC12 cells were cultured in poly-D-lysine coated (0.05 mg/ml) T75 flasks in DMEM medium (10% HS, 1% FBS and 1 IU/ml penicillin and 1 μg/ml streptomycin). Approximately 70% of the medium was changed every second or third day. The cells were grown to 70–80% of confluence and split 1:3–4 using 0.5 mM EDTA solution in PBS (5 min incubation at 37 °C, 5% CO_2_ followed by gentle cell scraping). PC12 cells were induced to neuronal differentiation using nerve growth factor [4] essentially as described by Li et al. [46] with the following modifications. PC12 cells were differentiated in DMEM medium (1% HS, 1 IU/ml penicillin and 1 μg/ml streptomycin) freshly supplemented with 100 ng/ml rat NGFβ. The cells (500 cells/mm^2^) were plated in black clear bottom 96-well plates in the culture medium overnight. Next day, the medium was replaced with the differentiation medium. Every second day, half of the medium/well was changed.

SH-SY5Y cells were cultured in EMEM medium (10% FBS, 1% NEAA, 2 mM L-glutamine, 1 IU/ml penicillin and 1 μg/ml streptomycin) in T75 flasks. Medium was changed every third to fourth day. The cells were grown to 70–80% of confluence and split to 10,000 cells/cm^2^ or 25,000 cells/cm^2^. To passage the cells, 0.05% Trypsin/0.02% EDTA solution in PBS was applied for 5 min at 37 °C, 5% CO_2_. Differentiation of SH-SY5Y cells was performed with 1 μM RA in DMEM F12 (1:1) medium (1% N2, 1 IU/ml penicillin and 1 μg/ml streptomycin) [47]. The cells were plated at a density of 500 cells/mm^2^ in black clear bottom 96-well plates in the culture medium overnight. The day after, the medium was changed to the differentiation medium. Half of the medium/well was replaced every second day.

### Chemical exposure

The cell lines were plated at a density of 500 cells/mm^2^ in 96-well plates. MeHg and okadaic acid were dissolved in DMSO. The final concentration of DMSO in the samples was 0.1%. Acrylamide was dissolved in PBS. Buthionine sulfoximine (BSO) and reduced glutathione (GSH) were dissolved in Milli-Q water. On day 6 in the differentiation media, the cells were exposed to MeHg (0.05–1 μM), okadaic acid (0.5–10 nM) and acrylamide (0.1–1000 μM) for 48 h. To investigate the importance of glutathione status on MeHg-induced toxicity, cells were pretreated with BSO (100 μM) for 17 h or GSH (1 mM) for 1 h prior to MeHg (1 μM) exposure for 24 h.

### Cell viability analyses

Calcein-AM is a fluorescence-based cell viability assay. The non-fluorescent compound calcein-acetoxymethyl (AM) enters the cells where intracellular esterases remove the AM group. The fluorescent calcein is retained in the living cells, and it can be detected at the excitation/emission maximum of 495/550 nm [48, 49]. Samples were washed with PBS and incubated with 1 μM calcein-AM for 1 h at room temperature protected from light. The fluorescence was measured in BMG FLOUstar Galaxy microplate reader (BMG Lab technologies, Offenburg, Germany) with 490/520 nm excitation/emission filters. Representative images of the samples were taken with a Nikon Eclipse TE2000-U inverted microscope with a Plan Fluor ELWD 20×/0.45 objective with a Nikon Digital Sight DS-5Mc camera (Tekno Optik AB, Skärholmen, Sweden). Samples treated with 2% Triton X-100 for 30 min at 37 °C, 5% CO_2_ served as controls for maximal cell death.

Cellular metabolic reduction was measured with PrestoBlue™ resazurin-based assay. PrestoBlue reagent (10 μL/well) was added to 100 μl of cells in media. After 30 min of incubation at 37 °C, 5% CO_2_, fluorescence was measured in the FLUOstar Galaxy plate reader with 544/612 nm excitation/emission filters. LDH activity in cell culture media that indicate damage to the cell membrane was assessed with LDH cytotoxicity detection kit according to the manufacturer’s instructions. Cell culture media (100 μl/well) were transferred to a clear 96-well plate and 100 μl of the kit reagent mixture was added. After 30 min of incubation at room temperature, absorbance was measured at 490 nm (reference wavelength 650 nm) in the SPECTROstar Nano microplate reader (BMG LABTECH GmbH, Offenburg, Germany). The media from cells treated with 2% Triton X-100 solution for 30 min at 37 °C, 5% CO_2_ were used to detect the maximal LDH content in the medium.

### Immunofluorescence detection of βIII-tubulin

The samples were fixed in 4% formaldehyde for 30 min, permeabilized with 0.1% Triton X-100 for 5 min, and washed three times with PBS. To minimize unspecific antibody binding, blocking solution (3% FBS in PBS) was applied for 30 min. The purified polyclonal antibodies against βIII-tubulin diluted 1:500 in 3% FBS were added to the cells for 1 h incubation. The samples were washed three times with PBS, and the secondary antibodies Alexa Fluor 488, diluted 1:250 in 3% FBS, were applied for 1 h. After three washes in PBS, fluorescence in the samples was measured in the FLOUstar Galaxy microplate reader with 485/520 nm excitation/emission filters. Representative images of the samples were obtained using a Nikon Eclipse TE2000-U inverted microscope with a Plan Fluor ELWD 20x/0.45 objective with a Nikon Digital Sight DS-5Mc camera. The samples exposed to 2% Triton X-100 for 30 min at 37 °C, 5% CO_2_ were used as controls of the minimal amount of fluorescence in dead cells.

### Mitochondrial membrane potential (∆Ψm) analysis

Alterations in mitochondrial membrane potential was measured with TMRE (tetramethylrhodamine ethyl ester) assay. TMRE is a positively charged dye that accumulates in negatively charged mitochondria. Less dye accumulates in inactive or depolarized mitochondria due to their less negative charge compared to polarized or active mitochondria [50]. TMRE (1 mM) stock solution was prepared in DMSO and stored at −20 °C. The stock solution was diluted in the cell culture media to the working solution of 5 μM TMRE. In the cell samples, the medium was changed and TMRE was added to the final concentration of 500 nM for 30–45 min incubation at 37 °C, 5% CO_2_. The cells were washed once with 100 μl of HBSS containing 0.2% bovine serum albumin (BSA), and 200 μl of HBSS/0.2% BSA was added to the samples. Fluorescence was measured in the FLUOstar Galaxy plate reader with 544/590 nm excitation/emission filters.

### Statistical analysis

Three to six independent experiments were performed for each assay with triplicates or duplicates for each experimental condition. Statistical analyses (one- or two-way ANOVA for repeated measures with Dunnett’s or Bonferroni’s *post hoc* multiple comparisons tests) were undertaken in the GraphPad Prism computer program for the Macintosh, version 6 (GraphPad Software Inc., San Diego, CA, USA).

## Results

### Neuronal differentiation of the P19, PC12 and SH-SY5Y cell lines

**Fig. 1:**
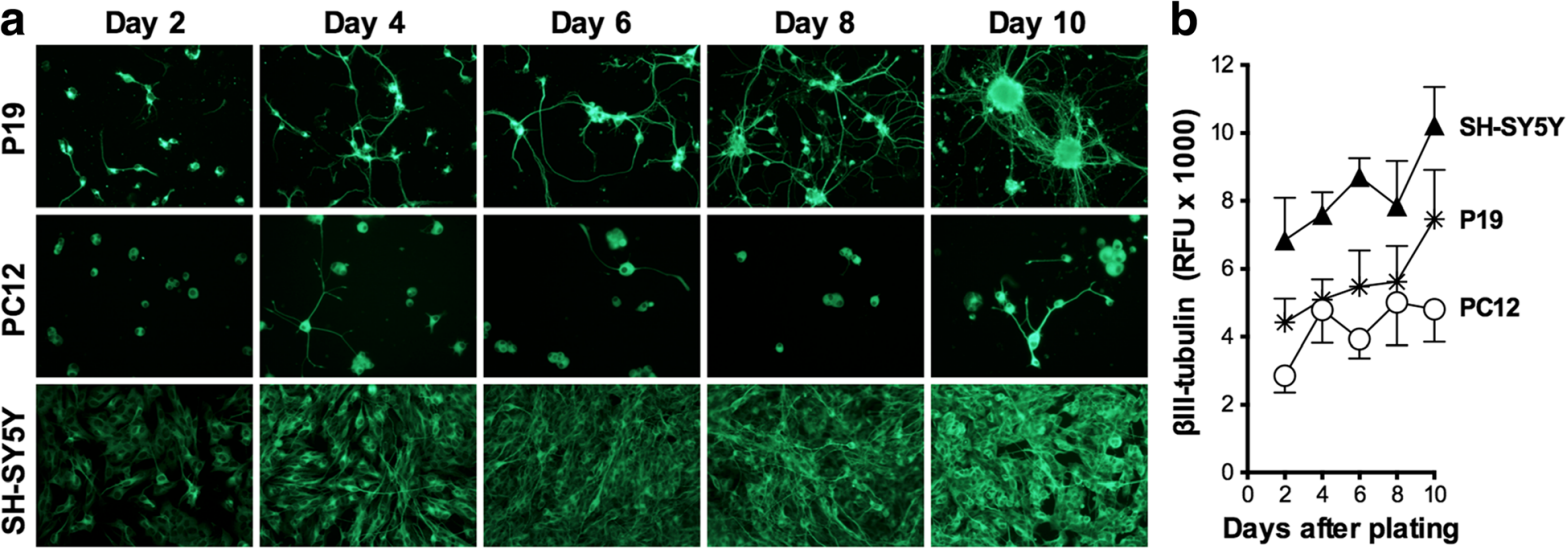
Development of neurons derived from RA-treated P19 and SH-SY5Y cells, and NGF-stimulated PC12 cells up to 10 days in culture. The cells were plated at a density of 500 cells/mm^2^ and immunostained against the neuron-specific protein βIII-tubulin. **a** Representative fluorescence microscopy images of neurons (20 × magnification). **b** Fluorescence of anti-βIII-tubulin antibodies measured in a microplate reader and expressed as relative fluorescence units (RFU). Data are means ± SEM of 3–4 independent experiments

The process of neurite outgrowth during differentiation (days 2–10) was assessed using immunostaining against βIII-tubulin. The representative images of the cells are shown in Fig. 1a, and the fluorescence intensities (expressed in relative fluorescence units) of the secondary antibodies bound to anti-βIII-antibodies are present in Fig. 1b. RA-treated P19 cells showed the most complex neuronal network, with neurite elongation and branching, among the three cell models (Fig. 1a). The amount of βIII-tubulin fluorescence increased as the process of neuronal differentiation proceeded in P19 and SH-SY5Y cells and to a lesser extent in PC12 cells. The P19 cells did not proliferate in the serum-free differentiation medium, and the increase in the amount of βIII-tubulin fluorescence was due to an increase in neurite extensions [51]. SH-SY5Y cells continued to proliferate during the process of differentiation. Therefore, the increase in βIII-tubulin fluorescence was due to the increasing number of cells in addition to neurite extensions. Less number of PC12 cells possessed neurites compared to the other two models explaining lower increase in βIII-tubulin fluorescence (Fig. 1a and b).

### Effects of MeHg, okadaic acid and acrylamide upon neuronal viability

**Fig. 2:**
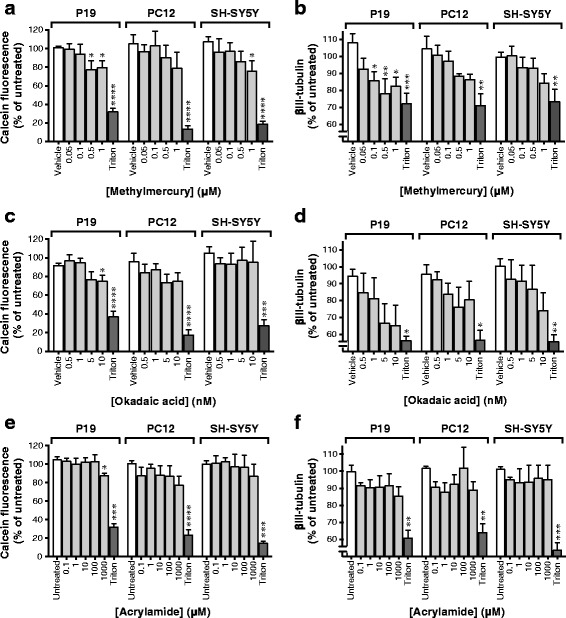
Concentration-dependent effects of MeHg, okadaic acid and acrylamide on cell viability and expression of the neuron-specific protein βIII-tubulin in neuronally differentiated P19, PC12 and SH-SY5Y cells. The cells were plated at a density of 500 cells/mm^2^ and cultured for 6 days in the differentiation media, followed by exposure to the test compounds for 48 h. Effects of MeHg (**a**, **b**), okadaic acid (**c**, **d**) and acrylamide (**e**, **f**) on the cell viability were assessed by using the calcein-AM assay (**a**, **c**, **e**), and the immunofluorescence of βIII-tubulin (**b**, **d**, **f**). The data are means ± SEM of *n* = 6 independent experiments (*n* = 3 for okadaic acid in the βIII-tubulin assay; panel **d**). The results are expressed as percentage of non-treated cells or cells treated with 0.1% DMSO (used as vehicle). Wells treated with 2% Triton X-100 for 30 min served as controls for maximal cell death. Statistical analysis was performed using repeated measures one-way ANOVA with post hoc Dunnett’s multiple comparisons test (*p < 0.05, **p < 0.01, ***p < 0.001, and ****p < 0.0001) compared to corresponding controls

Differentiated P19 cells were more sensitive towards the toxicity produced by MeHg, okadaic acid and acrylamide compared to PC12 cells and SH-SY5Y cells, as assessed by calcein-AM assay and immunofluorescence detection of βIII-tubulin (Fig. 2). MeHg produced a concentration-dependent toxicity in the P19 neurons, with statistically significant effects at concentrations from 0.5 μM and higher in the calcein-AM assay, and from 0.1 μM and higher in the βIII-tubulin assay. Fluorescence (expressed as % of controls) seen following treatment with 0.5 μM of MeHg in the P19 neurons was 77 ± 10% (means ± SEM; Fig. 2a) and 78 ± 9% (Fig. 2b) for the calcein-AM and βIII-tubulin methodologies, respectively. In P12 cells, corresponding treatment showed 90 ± 13% and 88 ± 1%, and in SH-SY5Y cells 86 ± 11% and 93 ± 6%, respectively. There was a tendency for concentration-dependent toxic effects of MeHg in the PC12 cells and the SH-SY5Y cells, but the only statistically significant effect was obtained in the SH-SY5Y cells at a concentration of 1 μM, when measuring the fluorescence of calcein (*p* < 0.05). However, a two-way ANOVA of all MeHg data showed that MeHg produced a statistically significant decrease in the fluorescence of calcein (*p* < 0.001) and βIII-tubulin (*p* < 0.01), independently of the cell type examined (no interaction between concentrations of MeHg × cell type).

The potent cytotoxin okadaic acid, at a concentration of 10 nM, produced a significant reduction in cytosolic fluorescence of calcein in the P19 neurons (75 ± 6% of untreated controls; *p* < 0.05) (Fig. 2c). One-way ANOVA (with *post hoc* Dunnett’s multiple comparisons test) of the okadaic acid data revealed no other statistically significant effects. However, a two-way ANOVA of the same data showed that okadaic acid produced a statistically significant (*p* < 0.001) decrease in the fluorescence of anti-βIII-tubulin, independent of cell type.

**Fig. 3:**
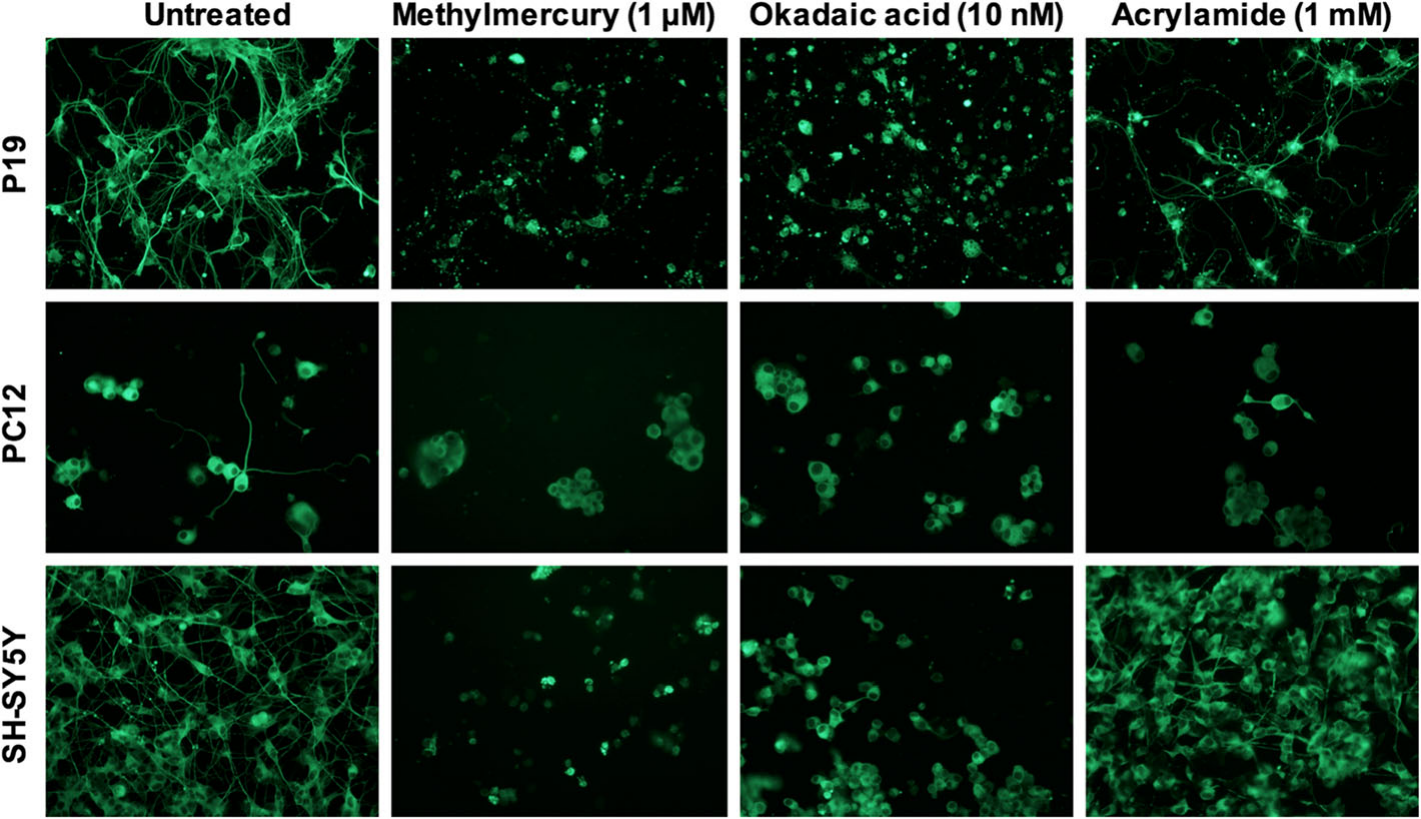
Representative fluorescence microscopy images of neuronally differentiated P19, PC12 and SH-SY5Y cells exposed to 1 μM methylmercury, 10 nM okadaic acid and 1 mM acrylamide. The cells were plated at a density of 500 cells/mm^2^ and cultured for 6 days in the differentiation media, followed by exposure to the test compounds for 48 h. The cells were immunolabeled against the neuron-specific protein βIII-tubulin and the fluorescence microscopy images were obtained at 20 × magnification

Acrylamide did not significantly affect the viability of the examined cell lines, except in the P19 neurons where 1 mM acrylamide reduced the fluorescence of calcein to 87 ± 3% of untreated controls (Fig. 2e; *p* < 0.05). Representative images of the cells exposed to MeHg (1 μM), okadaic acid (10 nM) and acrylamide (1 mM) immunostained against βIII-tubulin are shown in Fig. 3. In the fluorescence microscopy images, exposure of differentiated P19, PC12 and SH-SY5Y cells to 1 μM MeHg and 10 nM okadaic acid destroyed the neurites. Acrylamide (1 mM) partially damaged the neurites in P19 and PC12 cells, and to a lesser extent in SH-SY5Y cells (Fig. 3). The images show the examples of the cells labeled against βIII-tubulin and treated with the compounds, and do not provide a quantitative information about the effects of the chemicals on these cells. Fluorescence plate reader measurements of the cells shown in Fig. 2 quantify the effects of the chemicals on the fluorescence of anti-βIII-tubulin antibodies.

### Involvement of GSH in the MeHg-induced toxicity in differentiated P19 and SH-SY5Y cells

**Fig. 4:**
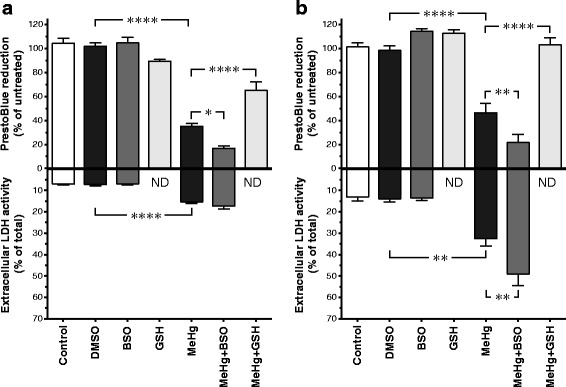
Effects of MeHg, BSO and GSH on the viability of RA-treated P19 cells (**a**) and SH-SY5Y cells (**b**). Cells cultured for 6 days in the differentiation media were pre-treated with 100 μM BSO for 17 h or 1 mM GSH for 1 h followed by exposure to 1 μM MeHg for 24 h. Cell viability was assessed with the PrestoBlue assay that measures cellular metabolic reduction, and extracellular LDH activity assay. Data are means ± SEM of *n* = 6 independent experiments. For the PrestoBlue assay, data are expressed as percentage of non-treated or 0.1% DMSO vehicle-treated cells. For the LDH assay, the data are presented as percentage of total cell death (cells treated with 2% Triton X-100). Statistical analysis was undertaken using one-way ANOVA with post hoc Bonferroni’s multiple comparisons test: *p < 0.05, **p < 0.01, and ****p < 0.0001 (the effect of the treatment compared to the corresponding vehicle control, or the effect of the combination of MeHg + GSH or MeHg + BSO compared to MeHg *per se*). ND = not determined

**Fig. 5:**
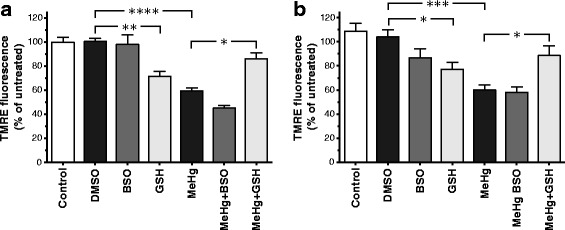
The effects of MeHg, GSH and BSO on TMRE fluorescence in RA-treated P19 (**a**) and SH-SY5Y cells (**b**). Cells cultured for 6 days in the differentiation media were pre-treated with 100 μM BSO for 17 h or with 1 mM GSH for 1 h followed by exposure to 1 μM MeHg for 24 h. Alterations in mitochondrial membrane potential were measured with the TMRE assay. Data are means ± SEM of *n* = 6 independent experiments, and expressed as percentage of non-treated or 0.1% DMSO vehicle-treated cells. Statistical analysis was undertaken using one-way ANOVA with post hoc Bonferroni’s multiple comparisons test: *p <0.05, **p < 0.01, ***p < 0.001, and ****p < 0.0001 (the effect of the treatment compared to the corresponding vehicle control, or the effect of the combination of MeHg + GSH or MeHg + BSO compared to MeHg *per se*)

To investigate if the toxicity produced by MeHg is affected by alterations of intracellular glutathione status, differentiated P19 and SH-SY5Y cells were preincubated with GSH or BSO. In P19 neurons and differentiated SH-SY5Y cells, preincubation with GSH reduced the MeHg toxicity as measured with both the PrestoBlue (Fig. 4) and the TMRE assays (Fig. 5). MeHg, at a concentration of 1 μM, produced a major statistically significant decrease in the cellular metabolic activity (35 ± 2% of vehicle controls in P19 neurons and 47 ± 8% in SH-SY5Y cells), with a simultaneous increase in extracellular LDH activity from 7 ± 1% (of total LDH content) in P19 controls to 15 ± 1%, and from 13 ± 2% in SH-SY5Y controls to 32 ± 3% (Fig. 4). Similar results were obtained in the TMRE assay (Fig. 5) where 1 μM MeHg reduced ∆Ψm to 59 ± 2% of controls in the P19 neurons and to 60 ± 4% in the SH-SY5Y cells. Preincubation with 1 mM GSH for 1 h attenuated the MeHg-induced decrease in PrestoBlue reduction and TMRE fluorescence in both cell lines (by approximately 30% or more in all assays). Exposure to 1 mM GSH for 25 h significantly decreased TMRE fluorescence in both cell models. In the treated P19 neurons and retinoic acid-differentiated SH-SY5Y cells, the TMRE fluorescence was 72 ± 4% and 77 ± 6%, respectively. The detected decrease in mitochondrial membrane potential might have been the consequence of the reductive stress produced by the excessive GSH levels in the cells [52]. No valid data of the effect of GSH in the LDH assay could be obtained, since GSH seems to directly interfere with the LDH test readout by producing abnormally high absorbance values. Preincubation with 100 μM BSO for 17 h significantly enhanced the MeHg-induced effects on cellular metabolic activity in both cell lines (to 16 ± 2% of P19 controls and 22 ± 7% of SH-SY5Y controls; Fig. 4), and the extracellular LDH activity in the SH-SY5Y cultures increased to 49 ± 5%. No statistically significant effects of BSO on the MeHg-induced decrease in mitochondrial membrane potential could be observed (Fig. 5).

## Discussion

With an increasing number of chemicals in the environment, there is a need for simple and robust in vitro cellular models for detection of their potential toxic effects. The purpose of this work was to evaluate the sensitivity of three different neuronal cell models, towards detection of chemical-induced neurotoxicity. Neurons derived from P19 cells were the most sensitive model in the neurotoxicity assessment of MeHg, okadaic acid and acrylamide. Decrease in intracellular fluorescence of calcein and amount of anti-βIII-tubulin immunofluorescence upon the exposure to the compounds was detected at lower concentrations in P19 neurons than in differentiated SH-SY5Y and PC12 cells.

The SH-SY5Y human neuroblastoma cell line has been long used as an in vitro neuronal model in neuroprotection and Parkinson’s disease research mainly due to its human origin and catecholaminergic properties [53]. The rat PC12 cells have been widely used to study the process of neuronal differentiation and regulation of neurite development [54, 55], but also to study vesicle function and exocytosis [56]. Previous studies have shown that both PC12 cells and SH-SY5Y cells are vulnerable to MeHg-induced neurotoxicity upon exposure in the beginning of the differentiation processes. In RA-treated SH-SY5Y cells, MeHg was toxic at lower concentrations when co-introduced with RA in the start of the differentiation process than after 2 days of differentiation [57]. In NGF-treated PC12 cells, MeHg reduced cell viability and inhibited neurite outgrowth at the initiation of the differentiation process at the low micromolar concentrations [2].

We found that the neuronally differentiated P19 cells is a more robust and sensitive model to detect MeHg-induced cytotoxicity compared to differentiated PC12 and SH-SY5Y cells. The mouse P19 cells have been used for many types of studies on neuronal development. A major advantage in neurotoxicology studies is that the P19 cells can differentiate into a wide range of neuronal and glial cell types with robust expression of GABAergic and glutamatergic phenotypes and the ability to form functional synapses [14] in contrast to most commonly used cell lines. The tumorigenic background of the P19 cell has been, however, considered a disadvantage compared to, for example, human stem cells. However, it is less expensive and time-consuming to maintain and differentiate P19 cells to neurons compared to human stem cells [58].

The known targets of MeHg toxicity include the glutathione system [25] and the mitochondria [27]. We showed that extracellular GSH exposure reduced MeHg-induced toxicity in both RA-treated P19 cells and SH-SY5Y cells. BSO, the potent inhibitor of GSH synthesis, enhanced the negative effect of MeHg upon cellular metabolic activity. The results are in line with the study of Sanfeliu et al. [24] in which GSH and BSO exposure had similar effects on the toxicity of MeHg in human neurons, astrocytes and SH-SY5Y cells. Although, in our study, treatment with extracellular GSH alone decreased mitochondrial membrane potential that might have been caused by the reductive stress that affected mitochondria [52]. In the study of Zhang et al. [52], exposure of the embryonic rat myocardial cell line H9c2 to *N*-acetyl-_*L*_-cysteine (NAC), the precursor of GSH, led to increased levels of GSH, mitochondrial oxidation and cytotoxicity. Singh et al. [59] demonstrated that 24 h treatment of the rat skeletal muscle cell line L6 with 1 mM NAC did not affect cell viability but increased production of superoxides that are damaging to cells. However, in the study on different mammalian cell lines, treatment with NAC and GSH ethyl ester, caused a rapid mitochondrial oxidation that did not involve alterations in mitochondrial membrane potential measured with the TMRE probe [60].

Okadaic acid has been shown to inhibit neurite outgrowth in NGF-treated PC12 cells at low nanomolar concentrations [61], and in cultured rat cortical neurons and the human neuroblastoma cell line MSN, okadaic acid induced changes in neuronal cytoskeleton that led to cell death [62]. In the present study, okadaic acid showed a tendency to concentration-dependently reduced the fluorescence of anti-βIII-tubulin in all cell types, but the great variance of the data precluded from obtaining statistical significance. However, in the P19 neurons, at a concentration of 10 nM, okadaic acid showed a statistically significant reduction in the fluorescence of calcein. This enlightens the importance of using several different methods to assess the neurotoxicity of chemicals which often in dose-response studies show what could be described as threshold effects.

Acrylamide significantly reduced cell viability in P19 cells at the concentration of 1 mM, but not in the PC12 or SH-SY5Y cells. Although, in the study of Attoff et al. [3] acrylamide applied to SH-SY5Y cells in the beginning of the differentiation process for 3–6 days greatly reduced cell proliferation at the same concentration. Additionally, the same study reported that acrylamide impaired neurite outgrowth at the concentration of 100 nM after 3 days of exposure. That might indicate that SH-SY5Y cells are more susceptible to acrylamide neurotoxicity at the early stage of differentiation and/or at longer times of exposure than examined in the present study. In NGF (50 ng/ml)-stimulated PC12 cells, Chen et al. [63] showed that acrylamide inhibited cells proliferation at the concentration of 0.5 mM, and at 0.1 mM reduced neurite outgrowth after 48 h of exposure. The exposure to acrylamide was performed directly at the start of the differentiation process along with NGF treatment. Therefore, acrylamide might be more toxic when applied at the early stage of cell differentiation compare to the later stage assessed in our study.

## Conclusions

The P19 neurons were more sensitive to detect general cytotoxicity of MeHg, okadaic acid and acrylamide than the PC12 cells and the SH-SY5Y cells during neuronal differentiation. Additionally, P19 neurons had the most complex structure of neuronal network compared to SH-SY5Y and PC12 cells. P19 neurons were also able to detect specific targets of MeHg toxicity as the GSH system and the mitochondria. Therefore, the P19 neuronal model may have an application as an in vitro cell model for screening of chemicals for neurotoxic effects and for evaluation of specific mechanism of toxicity especially during the process of neuronal development. The main limitation of the model includes the mouse carcinoma origin. The advantages include a simple and robust model with many properties of mammalian brain cells in culture, that is less expensive to maintain than human stem cells.

## Abbreviations

BSO: Buthionine sulfoximine
calcein-AM: Calcein-acetoxymethyl
GABA: Gamma-aminobutyric acid
GSH: Glutathione
LDH: Lactate dehydrogenase
MeHg: Methylmercury
NAC: *N*-acetyl-_L_-cysteine
NGF: Nerve growth factor
RA: Retinoic acid
TMRE: Tetramethylrhodamine ethyl ester
